# Postoperative complications and mortality following emergency digestive surgery during the COVID-19 pandemic: A multicenter collaborative retrospective cohort study protocol (COVID-CIR)

**DOI:** 10.1097/MD.0000000000024409

**Published:** 2021-02-05

**Authors:** Zoilo Madrazo, Javier Osorio, Aurema Otero, Sebastiano Biondo, Sebastian Videla

**Affiliations:** a Department of General and Digestive Surgery, Bellvitge University Hospital; b Clinical Research Support Unit, Clinical Pharmacology Department, Bellvitge University Hospital/Bellvitge Biomedical Research Institute (IDIBELL); c Department of Pathology and Experimental Therapeutics, Faculty of Medicine, Universitat de Barcelona, L’Hospitalet de Llobregat, Barcelona, Spain; d Investigators and Co-Investigators from Participant Hospitals (see Supplementary data file 1).

**Keywords:** accident & emergency surgery, COVID-19, gastroenterology, surgery

## Abstract

Infection with the SARS-CoV-2 virus seems to contribute significantly to increased postoperative complications and mortality after emergency surgical procedures. Additionally, the fear of COVID-19 contagion delays the consultation of patients, resulting in the deterioration of their acute diseases by the time of consultation. In the specific case of urgent digestive surgery patients, both factors significantly worsen the postoperative course and prognosis. Main working hypothesis: infection by COVID-19 increases postoperative 30-day-mortality for any cause in patients submitted to emergency/urgent general or gastrointestinal surgery. Likewise, hospital collapse during the first wave of the COVID-19 pandemic increased 30-day-mortality for any cause. Hence, the main objective of this study is to estimate the cumulative incidence of mortality at 30-days-after-surgery. Secondary objectives are: to estimate the cumulative incidence of postoperative complications and to develop a specific postoperative risk propensity model for COVID-19-infected patients.

A multicenter, observational retrospective cohort study (COVID-CIR-study) will be carried out in consecutive patients operated on for urgent digestive pathology. Two cohorts will be defined: the “pandemic” cohort, which will include all patients (classified as COVID-19-positive or -negative) operated on for emergency digestive pathology during the months of March to June 2020; and the “control” cohort, which will include all patients operated on for emergency digestive pathology during the months of March to June 2019. Information will be gathered on demographic characteristics, clinical and analytical parameters, scores on the usual prognostic scales for quality management in a General Surgery service (POSSUM, P-POSSUM and LUCENTUM scores), prognostic factors applicable to all patients, specific prognostic factors for patients infected with SARS-CoV-2, postoperative morbidity and mortality (at 30 and 90 postoperative days). The main objective is to estimate the cumulative incidence of mortality at 30 days after surgery. As secondary objectives, to estimate the cumulative incidence of postoperative complications and to develop a specific postoperative risk propensity model for SARS-CoV-2 infected patients.

The protocol (version1.0, April 20th 2020) was approved by the local Institutional Review Board (Ethic-and-Clinical-Investigation-Committee, code PR169/20, date 05/05/20). The study findings will be submitted to peer-reviewed journals and presented at relevant national and international scientific meetings.

ClinicalTrials.gov Identifier: NCT04479150 (July 21, 2020).

**Strengths and limitations of this study**

- The multicentric design of the study will allow the recording of a large number of operated patients.
- The specific emergency digestive pathology considered and the recording of clinical conditions and prognostic scores will reduce the heterogeneity of population included in this study.
- The impact of SARS-CoV-2 viral status, COVID-19-related prognostic factors, the validity of surgical prognostic scores and the specific characteristics of initial pandemic period in Spain (March–June 2020) on postoperative outcomes will be assessed.
- The control cohort (same period, 2019) will represent a proper comparative group.
- Due to the retrospective design of the study, and the very first contact of Spanish Health System with the COVID-19 at the beginning of the study period, some data may be lost.

## 1 Introduction

Since early 2020, the rapid spread of the pandemic due to the SARS-CoV-2 virus (coronavirus 2 of severe acute respiratory syndrome) has triggered a complex and profound global sanitary, social, and economic crisis.^[1–4]^ As of August 7th, 2020, the pandemic has infected more than 18.9 million people, with a devastating death toll of more than 709,500 worldwide (official data from the World Health Organization, WHO).^[2,5]^ Spain, with more than 314,300 confirmed cases and more than 28,500 deaths, constitutes one of most severely hit countries (official data as of 08/07/20 from the Ministry of Health, Consumption and Social Welfare, Spain).^[6,7]^ Multiple clinical guidelines, protocols and recommendations have been developed to standardize medical practice and protect both patients and professionals.^[8–11]^ All health specialties have adopted forceful contingency plans and a profound reorganization of their healthcare activity.^[7,11]^ Emergency digestive surgery has not been different.^[11–15]^ During the period of maximum impact of the pandemic, most scheduled surgical interventions were drastically reduced or suspended, operating rooms were converted into critical care units, and highly versatile surgical staff were redistributed to medical services.^[14,16–20]^ However, urgent and “non-delayable” interventions continued to be performed in patients who were occasionally infected with SARS-CoV-2.^[13,20–23]^

Some publications suggest that surgical procedures accelerate and exacerbate the clinical progression of COVID-19.^[17,24]^ Several teams have recently described classic emergency surgical pathologies with “atypical” forms of presentation and/or complicated postoperative evolution, differing from the usual “pre-pandemic” scenario.^[10,13,25–28]^

Urgent and emergency digestive interventions, extremely prevalent in our setting, are always associated with a high risk of complications and mortality, compared to elective surgeries.^[29,30]^ In the current context of the COVID-19 pandemic, in addition to the preoperative risk factors and the peculiarities inherent in all emergency surgery, other different elements seem to influence the worst postoperative prognosis, even in COVID-19-negative patients.^[21,31]^ Several factors may be involved:

1. patients’ fear of visiting the hospital during the pandemic (and/or home confinement) makes that clinicians face with more advanced surgical pathologies in the moment of consultation;
2. “saturation” of the health system, collapse of the emergency departments, and the misinterpretation of septic symptoms in this context can easily add an “extra” delay in diagnosis;
3. conservative treatment (nonsurgical management) may be forced beyond usual local standards, making patients who ultimately need a surgical rescue worse off;
4. work overload of supporting services, such as intensive care units and interventional radiology, may increase “failure to rescue” in complicated patients, not just COVID-19-positive patients.^[16,21–23,32–35]^

Furthermore, we can’t exclude a synergistic negative effect of SARS-CoV-2 infection on the acute disease that prompted surgical consultation.^[35]^

The COVID-19 pandemic seems to directly or indirectly modify the clinical profile of the urgent surgical pathology treated in a General Surgery Emergency Department (epidemiology, surgical indications, clinical severity, diagnostic delay, complications, etc.).^[21,36]^ The surgical indications and operative techniques for urgent digestive surgeries performed during the “pandemic” period may differ from the “pre-pandemic” period, as well as the postoperative complication and mortality rates of COVID-19-negative patients during the pandemic period.^[21,23,25,37]^

Digestive surgeons ask several questions in this particular epidemiological context:

1. To what extent does COVID-19 infection modify the nature, clinical profile, or postoperative evolution of urgent surgical pathology?;^[17,21,26]^
2. Does the socio-sanitary context of a pandemic modify the presentation, severity, diagnostic delay, or postoperative evolution of COVID-19-negative patients undergoing emergency surgery?;^[21,32]^
3. Do the usual postoperative risk prediction scales maintain their validity in this period or do they require any kind of adjustment based on other specific prognostic criteria?;^[38,39]^
4. Is it appropriate to perform emergency surgical interventions with the classic clinical indications or should some acute pathologies be deferred and the surgery be postponed until normal conditions prevail?;^[10,15,25,36,37]^
5. Are all the surgical techniques and approaches commonly used in emergency digestive surgery equally safe during COVID-19 pandemic period?^[9,23,33,36,37,40]^

These and other questions should be answered as soon as possible. The analysis of real data on urgent gastrointestinal surgery in patients operated on during this period could provide the necessary information.^[36,41]^ However, in order to control the confounding factors of the pandemic scenario, cohort studies should provide additional information on the pathology and clinical condition of patients at the time of urgent surgery, for example with prognostic surgical scores. The classic surgical prognostic scales validated for the estimation of postoperative risk (scores POSSUM-mortality, POSSUM-morbidity, P-POSSUM, LUCENTUM-logistic regression, and LUCENTUM-CHAID) may not be adequate in patients undergoing emergency surgery during the pandemic period.^[38,39,42–45]^ Therefore, it may be necessary to consider other prognostic factors and design a new and specific prediction model for patients infected with SARS-CoV-2.^[46,47]^ Currently, we only have extrapolated prognostic scores for the set of COVID-19-positive patients, with little data on the specific subgroup of surgical patients, and even less information on the postoperative evolution after urgent digestive surgery.^[15,21,22,48–51]^

It would also be advisable to compare a COVID-19-positive group of patients with a simultaneous COVID-19-negative group (both in the “pandemic period”) and with a “pre-pandemic” control group in the same hospitals.

A registry of these characteristics would provide firmer evidence on their clinical profile and would make it possible to adequately estimate patient's postoperative risk.^[46]^ The few studies published to date address these issues superficially, with unadjusted raw data and a low degree of statistical analysis and scientific evidence.^[10,35,52]^

To contribute to the clarification of these doubts, we propose a multicenter, observational study (“COVID-CIR” study), in which multiple digestive surgical teams from different areas of Spain severely affected by the COVID-19 pandemic will participate. Main working hypothesis: infection by COVID-19 increases postoperative 30-day mortality for any cause in patients submitted to emergency/urgent general or gastrointestinal surgery. Likewise, hospital collapse during the first wave of the COVID-19 pandemic increased 30-day mortality for any cause in COVID-19-non-infected patients who required emergency/urgent general or gastrointestinal surgery. Hence, the main objective of this study is to estimate the cumulative incidence of mortality at 30 days after surgery. Secondary objectives are: to estimate the cumulative incidence of postoperative complications and to develop a specific postoperative risk propensity model for COVID-19 infected patients.

The conclusions of this study could contribute to design a future action plan for similar epidemiological situations or subsequent outbreaks of SARS-CoV-2.^[20,41,53]^

## 2 Methods and analysis

This protocol is reported in accordance with the STROBE guideline.^[54]^

### 2.1 Study design

COVID-CIR is an observational, multicenter, cohort study of patients undergoing emergency/urgent gastrointestinal surgery during the period of maximum impact of the COVID-19 pandemic in Spain (from March 1st to June 30th, 2020).^[6,13]^

This study was registered in the ClinicalTrials.gov Protocol Registration and Results System (Identifier: NCT04479150, July 21st, 2020).

#### 2.1.1 Ethical issues

The study conforms to the stipulations of the Declaration of Helsinki. The protocol was approved by the local Institutional Review Board (Ethics and Clinical Investigation Committee of the Hospital Universitari de Bellvitge, code PR169/20, date approval 05/05/20) and a waiver for written informed consent was given, as no additional procedures or information will be required from participants beyond that which would normally take place as part of clinical care. The list of local Institutional Review Board members is available at https://bellvitgehospital.cat/es/investiga-con-nosotros/ceic/composicion (accessed on August 11th, 2020). The level of protection of confidentiality, in terms of protection of personal data as required by Spanish Law (Organic Law on Data Protection 3/2018), is also ensured. The study findings will be submitted to peer-reviewed journals and presented at relevant national and international scientific meetings.

### 2.2 Settings

A total of 30 surgical teams from different Spanish hospitals have been proposed to participate in this study (see Supplementary data file 1), which has been coordinated by the Department of General and Digestive Surgery of the Bellvitge University Hospital (L’Hospitalet de Llobregat, Barcelona, Spain). All hospitals participating are from Spain, with different surgical capabilities.^[55]^

### 2.3 Study population

Patients operated on for emergency/urgent gastrointestinal surgery constitute the target population. Table 1 shows the list of all the urgent gastrointestinal surgery procedures under study. There is no patient or public involvement in the design, conduct, reporting, or dissemination plans of the study.

- Inclusion criteria: ≥18 years, both genders, and operated on for urgent/emergency gastrointestinal pathology.
- Exclusion criteria: patients who have undergone a scheduled surgical intervention or patients operated on by a service other than General and Digestive Surgery.

**Table 1:** Urgent gastrointestinal surgical procedures according to the degree of surgical complexity.

• Minor Complexity
- Hernia/eventration
- Perianal surgery, pilonidal sinus
• Moderate Complexity
- Cholecystectomy (open or laparoscopic)
- Appendectomy (open or laparoscopic)
• Major Complexity
- Gastrointestinal perforation suture
- Intestinal resection
- Colectomy
- Main bile duct surgery
- Gastrectomy
- Lysis of adhesions or reduction of internal hernia or enterolithotomy
- Splenectomy or minor liver trauma
- Exploratory laparotomy
- Surgical hemostasis due to hemoperitoneum
• Major + Complexity
- Open pancreatic necrosectomy
- Pancreatectomy
- Damage control surgery (due to trauma, bleeding, ischemia or peritonitis)
• Others

Two cohorts will be defined:

1. Cohort A: consecutive patients who required emergency/urgent gastrointestinal surgery from March 1st to June 30th, 2020 (“first wave” of the COVID-19 pandemic in Spain). This cohort will be stratified by SARS-CoV-2 infection status (according to the RT-PCR [Reverse Transcription-Polymerase Chain Reaction] test, or serological detection of antibodies):^[8,11,14,17,46]^
  - COVID-19-positive patients (infected by SARS-CoV-2), and
  - COVID-19-negative patients (not infected by SARS-CoV-2).
2. Cohort B: consecutive patients who required emergency/urgent gastrointestinal surgery from March 1st to June 30th, 2019 (control cohort of a “pre-pandemic” period).

**Figure 1:**
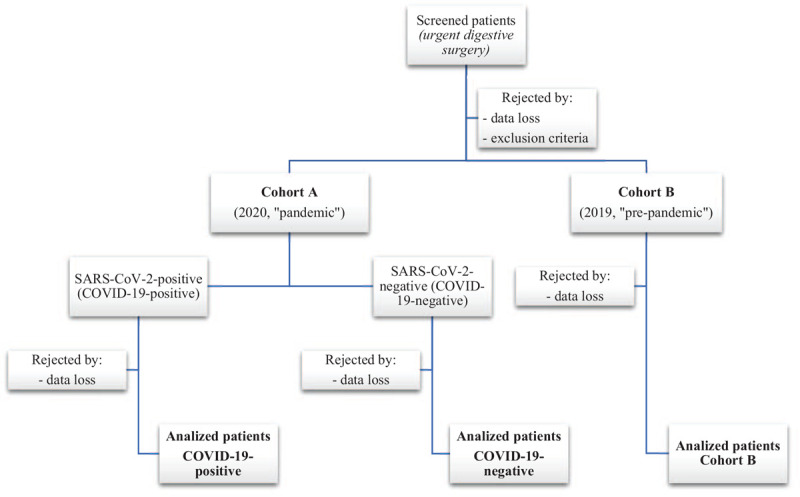
Flow diagram of the study.

Figure 1 shows the flow diagram of the study. Table 2 shows the complete list of postoperative complications and Table 3 shows the Clavien-Dindo classification for complications.^[56,57]^

**Table 2:** Listing and description of postoperative complications.

• Anastomotic dehiscence/intestinal fistula: clinical or radiological data of extravasation of intestinal content through an anastomosis, drainage, surgical wound, or abnormal hole
• Superficial wound dehiscence (full fascia)
• Evisceration (clinical or radiological; includes any degree)
• Heart failure or acute pulmonary edema: symptoms or signs of left or congestive ventricular insufficiency (change from preoperative situation)
• Fever of unknown origin: mantained fever >37°C for ≥24 hours, of unknown cause, after usual immediate postoperative temperature increase
• Mild hemorrhage: postoperative hemorrhage that does not require surgical re-examination or endoscopic/radiological procedure for treatment
• Severe bleeding: postoperative hemorrhage that does require surgical re-examination or endoscopic/radiological procedure for treatment
• Hypotension: maintained drop of systolic pressure to <90 mm Hg for >24 hours, detected with sphygmomanometer or arterial catheter
• Superficial wound infection: redness and pain around the surgical wound or local suppuration
• Deep wound infection: intra-abdominal collection (abscess), clinically or radiologically confirmed, or release of purulent content through a drainage
• Respiratory infection or pneumonia: purulent sputum with positive bacteriological/virological culture, with or without changes in chest X-ray, or fever with pulmonary radiological consolidation
• Urinary tract infection: urinary symptoms or fever, associated with sediment with bacteriuria/leukocyturia or positive urine culture
• Renal failure: sharp increase in creatinine to ≥2 mg/dl (≥177 μmol/L) in patients with normal prior renal function, or a sharp increase in creatinine (>50%) in patients with chronic kidney failure, or need for renal replacement therapy
• Respiratory failure: breathing difficulty requiring emergency ventilatory support, or PaO_2_ <60 mm Hg and PaCO_2_ >45 mm Hg breathing ambient air
• Bacteremia-Sepsis: positive blood culture
• DVT (deep vein thrombosis) and/or PE (pulmonary embolism): clinical suspicion, radiological confirmation by ECO-doppler, chest-computed tomography or ventilation/perfusion scan, or *postmortem* diagnosis
• Postoperative ileus
• Pleural effusion/pulmonary atelectasis
• Intestinal perforation
• Seroma or surgical wound hematoma
• Intestinal occlusion
• Ostomy complications (bleeding, retraction, infection, dermatitis, fistula, stoma necrosis)
• Blood glucose disturbances maintained >24 hours
• Atrial fibrillation
• Hypertensive crises (systolic blood pressure >200 mm Hg and/or diastolic blood pressure >120 mmHg)
• Acute confusion syndrome
• Gastrointestinal bleeding (upper or lower)
• Acute myocardial infarction, cerebrovascular accident or acute limb ischemia (peripheral artery ischemia)
• Acute mesenteric ischemia (intestinal ischemia, small bowel or colon)
• Cardiomyopathy or pericarditis

**Table 3:** Severity of postoperative complications according to Clavien-Dindo system.

Degree	Definition
I	Any deviation from the normal postoperative course that does not require surgical, radiological or endoscopic intervention. Includes additional use of electrolyte solutions, diuretics, antiemetics, antipyretics, analgesics and physiotherapy. Includes superficial infection or seroma/hematoma treated at the bedside.
II	Different pharmacological treatment than the above ones is required, including blood transfusions, antibiotics and total parenteral nutrition.
III	Requires surgical, endoscopic or radiological intervention:
a	No general anesthesia
b	Under general anesthesia
IV	Life-threatening complication requiring treatment in intermediate or intensive care unit:
a	Single organ dysfunction (includes haemodialysis)
b	Multiple organic dysfunction
V	Patient death

### 2.4 Study timeline

The study period has been delimited by the period of the “COVID-19 pandemic”: March 1st to June 30th, 2020. In Spain, this period corresponds to the maximum impact of the pandemic on health services (due to contingency plans and the reorganization of medical services) and society at large (due the “state of alarm” implemented by the Spanish government, which resulted in home confinement, restrictions on mobility and other preventive measures).^[6]^

Schedule the study protocol was drawn up in March and April 2020. The IRB (Institutional Review Board) approved the protocol on May 5, 2020 (code PR169/20, date 05/05/20).

At the moment of submitting this manuscript, participant recruitment is in progress. The inclusion of patients data does not end until August 31st, 2020. Provided the 30-day follow-up of the last included patient is completed by July– to August 2020, and that 1 month will be required for database cleaning and statistical analysis, it is expected that the first results (including ≤30-day morbidity/mortality) will be available in September to October 2020. Complete analysis (including ≤90-day mortality) will be available in November to December 2020. Table 4 depicts the study schedule.

**Table 4:** Study schedule.

		Follow-up		
Data	Enrollment^∗^	Day of hospital discharge/death	Day 30 of surgery	Day 90 of surgery
Inclusion/exclusion criteria	✓			
Day of surgery	✓			
Demographic data^†^	✓			
Clinical data^†^	✓			
Prognostic scores (see Table 5)	✓			
Presurgery evaluation	✓			
Urgent surgery characteristics (see Table 1)	✓			
Postsurgery evaluation: Mortality		✓	✓	✓
Postsurgery evaluation: Morbidity (see Table 2)		✓	✓	
In cohort 2020, COVID-19-positive:				
• RT-PCR or serological detection	✓			
In cohort 2020, COVID-19-positive:				
• COVID-19-related complications^‡^		✓	✓	

### 2.5 Outcomes

- The primary outcome is the cumulative incidence of mortality for any cause at 30 days after urgent gastrointestinal surgery.
- Secondary outcomes are:

1. Cumulative incidence of all-causes mortality within 90 days after surgery.
2. Cumulative incidence of any postoperative morbidity within 30 days after surgery: overall postoperative complications and/or complications specifically associated with the evolution of COVID-19 (Table 2).^[8,58]^
3. Cumulative incidence of severe 30 day-postoperative complications, >IIIA grade according to the Clavien-Dindo score (Table 3).^[56,57,59]^
4. Failure-to-rescue defined as the percentage of patients dying of any cause as a consequence of any postoperative complication.
5. Length of hospital stay (from the day of admission to the date of hospital discharge or death).
6. “Physiological” and “operative severity” to calculate the estimated risks of mortality and postoperative complications according to 5 surgical prognostic scales (Table 5): POSSUM-mortality and POSSUM-morbidity scores, P-POSSUM score, LUCENTUM-logistic regression, and LUCENTUM-CHAID scores.^[14,38,39,42–45]^
7. Attitude of the surgeons due to the influence of the SARS-CoV-2 pandemic regarding the “moment of surgical intervention”:^[21,22,32,33]^ Did it change? [No/Yes]; if affirmative:
  -Was the patient probably seen for consultation later than usual (more advanced pathology)?
  -Was the diagnosis of the surgical pathology delayed?
  -Was urgent intervention delayed for logistical problems (“hospital” delay)?
  -Was a conservative/non-operative treatment “over-indicated”?
8. Attitude of the surgeons due to the influence of the SARS-CoV-2 pandemic regarding the “surgical technique” used:^[9,10,15,36,37,40]^ Did it change? [No/Yes]; if affirmative:
  -Was the surgical technique being “more conservative than usual” or “more aggressive than usual”?

**Table 5:** Postoperative prognostic scores.

Score	Equation	Prediction
POSSUM (mortality)	Ln [R/(1–R)] = –7.04 + (0.13 × physiological score) + (0.16 × operative severity score)	Postoperative mortality
POSSUM (morbidity)	Ln [R/(1–R)] = –5.91 + (0.16 × physiological score) + (0.19 × operative severity score)	Postoperative morbidity
P-POSSUM	Ln [R/(1–R)] = –9.065 + (0.1692 × physiological score) + (0.155 × operative severity score)	Postoperative mortality
LUCENTUM-logistic regression	Ln [R/(1–R)] = –4.461 + (0.257 × age) + (0.261 × sodium) + (0.167 × Hb) + (0.364 × white cell count) + (0.397 × operative severity)	Postoperative morbidity
LUCENTUM-CHAID	Ln [R/(1–R)] = –5.835 + (0.757 × cardiac function) + (0.563 × sodium) + (0.411 × peritoneal soiling) + (0.778 × operative severity)	Postoperative morbidity

### 2.6 Data collection

All data will be gathered from electronic medical record. All consecutive patients operated on for urgent digestive surgery during the study period will be screened and included if they fulfil the inclusion criteria.

Demographic characteristics, comorbidities, clinical and analytical variables, SARS-CoV-2 infectious status (pre- and post-operative periods), clinical diagnosis, intraoperative findings and postoperative course (30-day morbidity, 30-day reinterventions, hospital stay, 30-day readmissions, and mortality at 30 and 90 days) will be recorded (Supplementary data file 2).^[25,35]^

All values necessary for the automatic calculation of the surgical prognostic scores shall also be included (Table 5).^[39,45]^

Prognostic factors recently described in COVID-19-positive patients will also be recorded: advanced age, hypertension, smoking, diabetes, chronic obstructive pulmonary disease (COPD), cardiovascular history, lymphocyte and platelet count, alanine-aminotransferase (ALT), C-reactive protein (CRP), D-dimer, ferritin, procalcitonin (PCT), lactate-dehydrogenase (LDH), troponin, prothrombin time, “neutrophil-to-lymphocyte ratio” (NLR) and “platelet-to-lymphocyte ratio” (PLR).^[8,11,16,26,35,49,50,60–64]^

Among postoperative complications, those currently considered “characteristics” of COVID-19 (pneumonia, respiratory failure, venous thromboembolism, acute myocardial infarction, cerebrovascular accident, peripheral arterial ischemia, intestinal ischemia, cardiomyopathy, or pericarditis) will be analyzed in depth.^[8,35,58]^

An electronic Case Report Form (eCRF), based on REDCap platform *(Research Electronic Data Capture software, REDCap Consortium)*, has been created *ad hoc* for this study (Supplementary data file 3).^[65]^

### 2.7 Data quality

Before closing the database for analysis, the data manager and the 2 principal investigators of the study (JO and ZM) will contact the senior local surgeon from each center in order to confirm completeness and accuracy of recorded data. Hospitals which failed to include all eligible patients of both periods will be excluded for analysis to avoid a selection bias. Hospitals failing to include at least 90% of operated patients fulfilling inclusion criteria during the study periods will be excluded for analysis in order to avoid selection bias. Moreover, patients with relevant missing information (age, sex, functional status, previous comorbidities, malignancy, COVID-19 infection status, date of surgery, urgency, type and complexity of surgery, and 30-day postoperative follow-up) will be also excluded.

### 2.8 Sample size

Due to the exploratory nature of our aim, no formal calculation of sample size was performed. It will be defined by the total number of patients who underwent emergency gastrointestinal surgery during the 2 study periods at the participating hospitals. We expect to include more than 2000 patients.

### 2.9 Statistical analysis

Baseline characteristics will be described using standard descriptive statistics, and a descriptive and exploratory comparative analysis between both cohorts will be carried out.

The cumulative incidence of mortality (and its 95% confident interval) and the incidence of morbidity (and its 95% confident interval) at 30 and 90 days will be estimated by positive/negative SARS-CoV-2 infection status (cohort A) and in the control group (cohort B).

To minimize the selection bias effect of potential confounders, propensity scores will be estimated using a multinomial model to estimate the probability that subjects were SARS-CoV-2-positive, SARS-CoV-2-negative or control. Subjects will be matched using the propensity scores. After matching, to identify imbalance between groups, the standardized mean difference will be estimated and plotted. In the matched sample, the cumulative incidence of mortality (and its 95% confidence interval) and the incidence of morbidity (and its 95% confidence interval) at 30 and 90 days will be compared using a generalized linear model with a binomial distribution and a logarithm link function. Those variables that remain imbalanced between groups after matching would be added to the model with an adjusting purpose.

A generalized linear model with a binomial distribution and a logarithm link function will be used to estimate relative risk as a measure to indentify prognostic factors associated with 30 and 90 days mortality and morbidity incidences. Five predictive scores of postoperative morbidity and/or mortality will be calculated according with Table 5: POSSUM-mortality, POSSUM-morbidity, P-POSSUM, LUCENTUM-logistic regression, and LUCENTUM-CHAID.^[38,39,42–45]^ Their applicability on postoperative morbidity or mortality prediction in COVID-19 pandemic will be assessed.

Data analysis will be carried out using R version 4.02 or superior (R Core Team, 2019; R: A language and environment for statistical computing. R Foundation for Statistical Computing, Vienna, Austria. URL https://www.R-project.org/).

## 3 Discussion

The COVID-19 worldwide pandemic, officially declared on March 11, 2020 by WHO, has drastically transformed the global socio-health landscape.^[2–5,8,66]^ Spain is one of the most affected countries by this unprecedented health crisis, ranking among the top nations in infected population, infected health workers, and deaths.^[6,13,55,67]^ All medical and surgical specialties have been closely affected.^[12,13,22,68,69]^

In the particular context of urgent surgery, SARS-CoV-2 virus infection appears to contribute to a significant increase in postoperative morbidity and mortality, higher than expected.^[22,25,27,35]^ Secondary “cytokine storm”, endothelial injury and microangiopathy, and the complex interaction of the virus with the immune system could explain (at least partially) this significant deterioration of the postoperative course described by several groups, especially in elderly patients and/or with associated diseases.^[8,11,26,35,58,60,61,70]^ Simultaneously, during the pandemic period, acute surgical pathologies attended in the emergency departments appear in more advanced stages and associate worse postoperative course, even in patients not affected by SARS-CoV-2.^[21–23,32,37]^

Risk scales (scores) for postoperative morbidity and/or mortality (POSSUM, P-POSSUM, and LUCENTUM scores) are essential for the outcomes of rigorous surgical audits, quality care controls and as a comparative framework (intra- or inter-hospital) of clinical outcomes.^[71]^ There is not information about the validity of these surgical predictive scores regarding urgent digestive surgery in COVID-19-positive or COVID-19-negative patients.^[14,39,45]^ Therefore, it is necessary to establish the predictive capacity of these scales and/or to select other significant prognostic factors in the COVID-19 pandemic period.^[35,41,46,48]^

This protocol has been designed to specifically assess the effect of the COVID-19 pandemic on patients (infected or not by SARS-CoV-2) operated on for some urgent digestive pathology (Table 1) during the period of maximum social and health impact of the disease in several hospitals in Spain.^[5,16,35]^ At the same time, the COVID-19-negative subgroup of patients in the 2020 cohort will be contrasted with the 2019 control group (“pre-pandemic”), in order to compare mortality rates and detect differences in clinical indications, surgical techniques used, and postoperative evolution between the 2 epidemiological contexts.^[15,33,36,37]^ The analysis of the data could provide us with useful information on the main predictors of postoperative complications or mortality of COVID-19-positive patients.^[41]^

Through an online electronic Case Report Form (eCRF) on REDCap platform,^[65]^ researchers from multiple Spanish hospitals will record the variables necessary to subsequently develop a comparison of the characteristics, postoperative morbidity, and mortality rates^[25,72]^ in 2 cohorts of patients operated on emergency digestive pathology: patients COVID-19-positive and COVID-19-negative operated during March to June 2020, and a control cohort of patients operated during the same period of the previous year (March–June 2019).^[23,25]^

As a secondary objective of the study, we are confident of obtaining sufficient data to develop our own model of postoperative risk prediction, specific to patients infected with SARS-CoV-2 (COVID-19-positive), as a useful short-, medium, and long-term clinical tool for clinical decision-making.^[52]^

This study has some limitation, including its inherent observational nature and the potential loss of perioperative analytical data (particularly, analytical parameters related to COVID-19 infection: CRP, D-dimer, ferritin, procalcitonin, LDH, and troponin), especially during the “pre-pandemic” period and the first weeks of the “pandemic period” (the beginning of health contact with the SARS-CoV-2 virus in Spain and/or absence of specific COVID-19 protocols or analytical profiles).

The information provided by this study could be a useful and extrapolable tool to surgical teams for urgent surgical decision-making on indications, surgical techniques, and resource management, both in the current pandemic context and in anticipation of future SARS-CoV-2 outbreaks.^[20,22,25,41,53]^

## Acknowledgments

We thank IDIBELL and CERCA Program/Generalitat de Catalunya for institutional support.

COVID-CIR Collaborative Group: The COVID-CIR Collaborative Group (in alphabetical last name order, with permission): Aurora Aldeano, Granollers General Hospital, Granollers; Eva Alonso, Cruces University Hospital, Bilbao; Martín Amarelo, Basurto University Hospital, Bilbao; Ainhoa Andrés, Donostia University Hospital, San Sebastian; Aitor Ariceta, Hospital Complex of Navarra, Pamplona; Lorena Arrabal, Donostia University Hospital, San Sebastian; Nares Arroyo, Granollers General Hospital, Granollers; Eva Artigau, Girona Dr. Josep Trueta University Hospital, Girona; Elisabet Baena, Bellvitge University Hospital, Barcelona; María Batllé, Granollers General Hospital, Granollers; Albert Caballero, Germans Trias i Pujol University Hospital, Badalona; Miguel Calle, Alto Deba Hospital, Mondragón, San Sebastián; Beatriz Campillo, Sant Joan de Deu Hospital Foundation, Martorell Hospital, Martorell; Andrea Campos, Parc Taulí Health Corporation, Sabadell Hospital, Sabadell; Aleidis Caro, Joan XXIII University Hospital, Tarragona; Ariadna Cidoncha, Parc Taulí Health Corporation, Sabadell Hospital, Sabadell; Arantxa Clavell, Germans Trias i Pujol University Hospital, Badalona; Pere Clos, Mataró Hospital, Maresme Health Consortium, Mataró; Pablo Collera, Althaia University Hospital, Xarxa Assistencial Universitària de Manresa, Sant Joan de Déu Hospital, Manresa; Natalia Cornellá, Bellvitge University Hospital, Barcelona; David Luis Coroleu, Barcelona Hospital, Barcelona; Javier Corral, Germans Trias i Pujol University Hospital, Badalona; Rafael Gerardo Díaz-del-Gobbo, Althaia University Hospital, Xarxa Assistencial Universitària de Manresa, Sant Joan de Déu Hospital, Manresa; Víctor Echenagusia, Araba University Hospital, Txagorritxu Hospital, Vitoria; Marina Esgueva, Cruces University Hospital, Bilbao; Roser Farré, Althaia University Hospital, Xarxa Assistencial Universitària de Manresa, Sant Joan de Déu Hospital, Manresa; Roser Flores, Althaia University Hospital, Xarxa Assistencial Universitària de Manresa, Sant Joan de Déu Hospital, Manresa; Miriam Flores, Granollers General Hospital, Granollers; Amador García-Ruiz-de-Gordejuela, Vall d’Hebron University Hospital, Barcelona; Alba García, Donostia University Hospital, San Sebastian; Eulogio Gardeazabal, Bidasoa Hospital, San Sebastián; Nico Garriga, Granollers General Hospital, Granollers; Elisenda Garsot, Germans Trias i Pujol University Hospital, Badalona; Marta Gil, Viladecans Hospital, Viladecans; Carlos Javier Gómez, Althaia University Hospital, Xarxa Assistencial Universitària de Manresa, Sant Joan de Déu Hospital, Manresa; Concepción Gómez, Vall d’Hebron University Hospital, Barcelona; Eneko González, Basurto University Hospital, Bilbao; Carmen González, Basurto University Hospital, Bilbao; Claudio Antonio Guariglia, Althaia University Hospital, Xarxa Assistencial Universitària de Manresa, Sant Joan de Déu Hospital, Manresa; Rosa Jorba, Joan XXIII University Hospital, Tarragona; Montserat Juvany, Granollers General Hospital, Granollers; Camilo Andrés López, Sant Joan Despí Moisès Broggi Hospital, Sant Joan Despí; Victoria Lucas, Parc Taulí Health Corporation, Sabadell Hospital, Sabadell; Eloy Maldonado, Girona Dr. Josep Trueta University Hospital, Girona; Lidia Martínez, Barcelona Hospital, Barcelona; Rodrigo Medrano, Sant Pau University Hospital, Barcelona; Robert Memba, Joan XXIII University Hospital, Tarragona; Estela Membrilla, Hospital del Mar University Hospital, Barcelona; Nuria Mestres, Arnau de Vilanova University Hospital, Lleida; Laura Millán, Dr. José Molina Orosa Hospital, Lanzarote; Joan Molins, Vic University Hospital, Vic; Alex Morera, Hospital del Mar University Hospital, Barcelona; Anna Muñoz, Parc Taulí Health Corporation, Sabadell Hospital, Sabadell; Ane Murúa, Basurto University Hospital, Bilbao; Esther Nve, Granollers General Hospital, Granollers; Carles Olona, Joan XXIII University Hospital, Tarragona; Jaume Ortega, Arnau de Vilanova University Hospital, Lleida; Alexander Leonel Osorio, Althaia University Hospital, Xarxa Assistencial Universitària de Manresa, Sant Joan de Déu Hospital, Manresa; Amalia Pelegrina, Hospital del Mar University Hospital, Barcelona; Silvia Pérez, Arnau de Vilanova University Hospital, Lleida; Noelia Pérez, Mútua de Terrassa University Hospital, Terrassa; María Pintado, Basurto University Hospital, Bilbao; Arantxa Rada, Granollers General Hospital, Granollers; Esther Raga, Sant Joan de Reus University Hospital, Reus; Araceli Rodríguez, Donostia University Hospital, San Sebastian; Patricia Ruiz-de-León, Granollers General Hospital, Granollers; David Ruiz, Terrasa Health Consortium, Terrassa Hospital, Terrassa; Beatriz Sáinz, Hospital Complex of Navarra, Pamplona; David Salazar, Igualada Hospital, Anoia Health Consortium, Igualada; Sergi Sánchez, Igualada Hospital, Anoia Health Consortium, Igualada; Raquel Sánchez, Althaia University Hospital, Xarxa Assistencial Universitària de Manresa, Sant Joan de Déu Hospital, Manresa; Lorena Sanchón, Althaia University Hospital, Xarxa Assistencial Universitària de Manresa, Sant Joan de Déu Hospital, Manresa; Maite Santamaría, Arnau de Vilanova University Hospital, Lleida; María José Sara, Hospital Complex of Navarra, Pamplona; Aingeru Sarriugarte, Cruces University Hospital, Bilbao; Cristina Soto, Althaia University Hospital, Xarxa Assistencial Universitària de Manresa, Sant Joan de Déu Hospital, Manresa; Jon Ignacio Uriarte, Basurto University Hospital, Bilbao; Marina Vila, Mataró Hospital, Maresme Health Consortium, Mataró; Ibabe Villalabeitia, Cruces University Hospital, Bilbao.

Biostatistics Unit (UBiDi, IDIBELL Foundation): Natalia Pallarés, Judith Peñafiel, Cristian Tebé.

## Author contributions

Concept and design: ZM, JO, SB. Drafting of the manuscript: ZM, JO (both authors contributed equally to the manuscript and share first authorship credit). Critical revision of the manuscript: AO, SV. All authors have read and approved the manuscript.

**Conceptualization:** Sebastian Videla, Zoilo Madrazo, Javier Osorio, Sebastiano Biondo.

**Data curation:** Zoilo Madrazo.

**Funding acquisition:** Sebastian Videla, Javier Osorio.

**Investigation:** Javier Osorio.

**Methodology:** Sebastian Videla, Aurema Otero.

**Supervision:** Zoilo Madrazo, Javier Osorio, Sebastiano Biondo.

**Writing – original draft:** Sebastian Videla, Zoilo Madrazo, Javier Osorio, Aurema Otero.

**Writing – review & editing:** Sebastian Videla, Zoilo Madrazo, Javier Osorio, Aurema Otero, Sebastiano Biondo.

## Supplementary Material

Supplemental Digital Content

## Supplementary Material

Supplemental Digital Content

## Supplementary Material

Supplemental Digital Content
