# Supplementary material for: Postoperative complications and mortality following emergency digestive surgery during the COVID-19 pandemic: A multicenter collaborative retrospective cohort study protocol (COVID-CIR)
Source: Medicine (Baltimore). 2021 Feb 5;100(5):e24409. doi: 10.1097/MD.0000000000024409 (PMC7870207; doi:10.1097/MD.0000000000024409)
Supplement: Supplemental Digital Content [file medi-100-e24409-s001.docx]

| **Hospital code (CNH)** | **hospital** | **principal investigator (PI) and co-investigators (Co-PI)** |
| --- | --- | --- |
| 080752 | Bellvitge University Hospital, Barcelona  (coordinating center) | Javier Osorio Aguilar (PI)  e-mail: [josorio@bellvitgehospital.cat](mailto:josorio@bellvitgehospital.cat)  Zoilo Madrazo González (PI)  e-mail: [zmadrazo@bellvitgehospital.cat](mailto:zmadrazo@bellvitgehospital.cat)  Elisabet Baena Sanfeliu (Co-PI)  e-mail: [ebaena@bellvitgehospital.cat](mailto:ebaena@bellvitgehospital.cat)  Natalia Cornellá Garceso (Co-PI)  e-mail: ncornella@bellvitgehospital.cat |
| 200261 | Donostia University Hospital,  San Sebastian | Araceli Rodríguez González (PI)  e-mail: [araceli.rodriguezgonzalez@osakidetza.eus](mailto:ARACELI.RODRIGUEZGONZALEZ@osakidetza.eus)  Ainhoa Andres Imáz (Co-PI)  e-mail: [ainhoa.andresimaz@osakidetza.eus](mailto:AINHOA.ANDRESIMAZ@osakidetza.eus)  Lorena Arrabal Agüera (Co-PI)  e-mail: [lorena.arrabalaguera@osakidetza.eus](mailto:lorena.arrabalaguera@osakidetza.eus)  Alba Garcia Trancho (Co-PI)  e-mail: alba.garciatrancho@osakidetza.eus |
| 080057 | Hospital del Mar University Hospital, Barcelona | Amalia Pelegrina Manzano (PI)  e-mail: [64144@parcdesalutmar.cat](mailto:64144@parcdesalutmar.cat)  Estela Membrilla Fernández (Co-PI)  e-mail: [94934@parcdesalutmar.cat‬‬‬‬‬](mailto:94934@parcdesalutmar.cat)  Alex Morera Grau (Co-PI)  e-mail: [64267@parcdesalutmar.cat‬‬‬‬‬](mailto:64267@parcdesalutmar.cat) |
| 080958 | Parc Taulí Health Corporation, Sabadell Hospital, Sabadell | Andrea Campos-Serra (PI)  e-mail: [acampos@tauli.cat](mailto:acampos@tauli.cat)  Anna Muñoz-Campaña (Co-PI)  e-mail: [amunozc@tauli.cat](mailto:amunozc@tauli.cat)  Ariadna Cidoncha-Secilla (Co-PI)  e-mail: [acidoncha@tauli.cat](mailto:acidoncha@tauli.cat)  Victoria Lucas-Guerrero (Co-PI)  e-mail: [vlucas@tauli.cat](mailto:vlucas@tauli.cat) |
| 480176 | Cruces University Hospital, Bilbao | Aingeru Sarriugarte Lasarte (PI)  e-mail: [aingeru.sarriugartelasarte@osakidetza.eus](mailto:aingeru.sarriugartelasarte@osakidetza.eus)  Eva Alonso Calderón (Co-PI)  e-mail: [eva.alonsocalderon@osakidetza.eus](mailto:eva.alonsocalderon@osakidetza.eus)  Marina Esgueva Angulo (Co-PI)  e-mail: [marina.esguevaangulo@osakidetza.eus](mailto:marina.esguevaangulo@osakidetza.eus)  Ibabe Villalabeitia Ateca (Co-PI)  e-mail: [ibabe.villalabeitiaateca@osakidetza.eus](mailto:ibabe.villalabeitiaateca@osakidetza.eus) |
| 080898 | Sant Joan de Deu Hospital Foundation, Martorell Hospital, Martorell | Beatriz Campillo Alonso (PI)  e-mail: bcampillo@hmartorell.es |
| 081326 | Mataró Hospital, Maresme Health Consortium, Mataró | Marina Vila Tura (PI)  e-mail: [Mvilatu@csdm.cat](mailto:Mvilatu@csdm.cat)  Pere Clos Ferrero (Co-PI)  e-mail: [pclos@csdm.cat](mailto:pclos@csdm.cat) |
| 081075 | Terrassa Health Consortium, Terrassa Hospital, Terrassa | David Ruiz Luna (PI)  e-mail: [druiz@cst.cat](mailto:druiz@cst.cat) |
| 081141 | Viladecans Hospital, Viladecans | Marta Gil Barrionuevo (PI)  e-mail: mgil.hv@gencat.cat |
| 250019 | Arnau de Vilanova University Hospital, Lleida | Maite Santamaría Gómez (PI)  e-mail: [mtsantamaria.lleida.ics@gencat.cat](mailto:mtsantamaria.lleida.ics@gencat.cat)  Núria Mestres Petit (Co-PI)  e-mail: [nmestres.lleida.ics@gencat.cat](mailto:nmestres.lleida.ics@gencat.cat)  Jaume Ortega Alcaide (Co-PI)  e-mail: [jaortega.lleida.ics@gencat.cat](mailto:jaortega.lleida.ics@gencat.cat)  Silvia Pérez Farré (Co-PI)  e-mail: [sperezf.lleida.ics@gencat.cat](mailto:sperezf.lleida.ics@gencat.cat) |
| 080863 | Althaia University Hospital, Xarxa Assistencial Universitària de Manresa-Sant Joan de Déu Hospital, Manresa | Carlos Javier Gómez Díaz (PI)  e-mail: [cjgomez@althaia.cat](mailto:cjgomez@althaia.cat)  Claudio Antonio Guariglia (Co-PI)  e-mail: [caguariglia@althaia.cat](mailto:caguariglia@althaia.cat)  Alexander Leonel Osorio Ramos (Co-PI)  e-mail: [alosorio@althaia.cat](mailto:alosorio@althaia.cat)  Lorena Sanchón Fructoso (Co-PI)  e-mail: [lsanchon@althaia.cat](mailto:lsanchon@althaia.cat)  Cristina Soto Montesinos (Co-PI)  e-mail: [csoto@althaia.cat](mailto:csoto@althaia.cat)  Rafael Gerardo Díaz del Gobbo (Co-PI)  e-mail: [rgdiaz@althaia.cat](mailto:rgdiaz@althaia.cat)  Roser Flores Clotet (Co-PI)  e-mail: [rfloresc@althaia.cat](mailto:rfloresc@althaia.cat)  Raquel Sánchez Jiménez (Co-PI)  e-mail: [rsanchezj@althaia.cat](mailto:rsanchezj@althaia.cat)  Roser Farré Font (Co-PI)  e-mail: [rfarre@althaia.cat](mailto:rfarre@althaia.cat)  Pablo Collera Ormazabal (Co-PI)  e-mail: pcollera@althaia.cat |
| 430017 | Joan XXIII University Hospital, Tarragona | Carles Olona Casas (PI)  e-mail: [colona.hj23.ics@gencat.cat](mailto:colona.hj23.ics@gencat.cat)  Aleidis Caro Tarragó (Co-PI)  e-mail: [acaro.hj23.ics@gencat.cat](mailto:acaro.hj23.ics@gencat.cat)  Robert Memba Ikuga (Co-PI)  e-mail: [rmembai.hj23.ics@gencat.cat](mailto:rmembai.hj23.ics@gencat.cat)  Rosa Jorba Martín (Co-PI)  e-mail: [rjorba.hj23.ics@gencat.cat](mailto:rjorba.hj23.ics@gencat.cat) |
| 081094 | Mútua de Terrassa University Hospital, Terrassa | Noelia Pérez Romero (PI)  e-mail: [nperez@mutuaterrasa.es](mailto:nperez@mutuaterrasa.es) |
| 170010 | Girona Dr.Josep Trueta University Hospital, Girona | Eva Artigau Nieto (PI)  e-mail: [eartigau.girona.ics@gencat.cat](mailto:eartigau.girona.ics@gencat.cat)  Eloy Maldonado Marcos (Co-PI)  e-mail: [emaldonadom.girona.ics@gencat.cat‬‬‬‬‬](mailto:emaldonadom.girona.ics@gencat.cat) |
| 080484 | Barcelona Hospital, Barcelona | David Luis Coroleu Lletget (PI)  e-mail: dcoroleu@scias.com  Lídia Martínez Fijo (Co-PI)  e-mail: lidiamafi87@hotmail.com |
| 310150 | Hospital Complex of Navarra, Pamplona | Beatriz Sainz Villacampa (PI)  e-mail: [mb.sainz.villacampa@navarra.es](mailto:mb.sainz.villacampa@navarra.es)  María José Sara Ongay (Co-PI)  e-mail: [mj.sara.ongay@navarra.es](mailto:mj.sara.ongay@navarra.es)  Aitor Ariceta Lopez (Co-PI)  e-mail: [aitor.ariceta.lopez@navarra.es‬‬‬‬‬](mailto:aitor.ariceta.lopez@navarra.es) |
| 010090 | Araba University Hospital, Txagorritxu Hospital, Vitoria | Victor Echenagusia Serrats (PI)  e-mail: victor.echenagusiaserrats@osakidetza.eus |
| 480078 | Basurto University Hospital, Bilbao | Carmen González Serrano (PI)  e-mail: [mariacarmen.gonzalezserrano@osakidetza.eus](mailto:mariacarmen.gonzalezserrano@osakidetza.eus)  Jon Ignacio Uriarte Teran (Co-PI)  e-mail: [jonignacio.uriarteteran@osakidetza.eus](mailto:JONIGNACIO.URIARTETERAN@osakidetza.eus)  Eneko Gonzalez Aguirregomezcorta (Co-PI)  e-mail: [eneko.gonzalezaguirregomezcorta@osakidetza.eus](mailto:ENEKO.GONZALEZAGUIRREGOMEZCORTA@osakidetza.eus)  Martin Amarelo Garcia (Co-PI)  e-mail: [martin.amarelogarcia@osakidetza.eus](mailto:MARTIN.AMARELOGARCIA@osakidetza.eus)  María Pintado Izquierdo (Co-PI)  e-mail: [maria.pintadoizquierdo@osakidetza.eus](mailto:MARIA.PINTADOIZQUIERDO@osakidetza.eus)  Ane Murua Ruiz (Co-PI)  e-mail: [ane.muruaruiz@osakidetza.eus](mailto:ane.muruaruiz@osakidetza.eus) |
| 080734 | Granollers General Hospital, Granollers | Aurora Aldeano Martín (PI)  e-mail: [aaldeanom@fphag.org](mailto:aaldeanom@fphag.org)  Nares Arroyo García (Co-PI)  e-mail: [narroyo@fphag.org‬‬‬‬‬](mailto:narroyo@fphag.org)  Maria Batlle Figueras (Co-PI)  e-mail: [mbatlle@fphag.org‬‬‬‬‬](mailto:mbatlle@fphag.org)  Miriam Flores Yélamos (Co-PI)  e-mail: [mflores@fphag.org‬‬‬‬‬](mailto:mflores@fphag.org)  Nico Garriga Rodríguez (Co-PI)  e-mail: [nngarriga@fphag.org‬‬‬‬‬](mailto:nngarriga@fphag.org)  Montserrat Juvany Gómez (Co-PI)  e-mail: [mjuvany@fphag.org‬‬‬‬‬](mailto:mjuvany@fphag.org)  Esther Nve Obiang (Co-PI)  e-mail: [enve@fphag.org‬‬‬‬‬](mailto:enve@fphag.org)  Arantxa Rada Palomino (Co-PI)  e-mail: [arada@fphag.org‬‬‬‬‬](mailto:arada@fphag.org)  Patricia Ruiz de León Muñoz (Co-PI)  e-mail: [pruizdeleon@fphag.org‬‬‬‬‬](mailto:pruizdeleon@fphag.org) |
| 081347 | Vall d’Hebrón University Hospital, General Surgery Department, Barcelona | Amador García Ruiz de Gordejuela (PI)  e-mail: [amador.garcia@vhebron.net](mailto:amador.garcia@vhebron.net) |
| 081347 | Vall d’Hebrón University Hospital, Hepatobiliopancreatic Surgery and Transplantation Department, Barcelona | Concepción Gómez Gavara (PI)  e-mail: concepcion.gomez@vhebron.net |
| 080667 | Germans Trias i Pujol University Hospital, Badalona | Arantxa Clavell Font (PI)  e-mail: [aclavell.germanstrias@gencat.cat](mailto:aclavell.germanstrias@gencat.cat)  Elisenda Garsot Savall (Co-PI)  e-mail: [egarsot.germanstrias@gencat.cat](mailto:egarsot.germanstrias@gencat.cat)  Albert Caballero Boza (Co-PI)  e-mail: [acaballero.germanstrias@gencat.cat](mailto:acaballero.germanstrias@gencat.cat)  Javier Corral Rubio (Co-PI)  e-mail: jcorral.germanstrias@gencat.cat |
| 080291 | Sant Pau University Hospital, Barcelona | Rodrigo Medrano Caviedes (PI)  e-mail: [rmedrano@santpau.cat](mailto:rmedrano@santpau.cat) |
| 082066 | Sant Joan Despí Moisès Broggi Hospital,  Sant Joan Despí | Camilo Andrés López Arévalo (PI)  e-mail: CamiloAndres.LopezArevalo@sanitatintegral.org |
| 081108 | Vic University Hospital, Vic | Joan Molinas Bruguera (PI)  e-mail: [jmolinas@chv.cat](mailto:jmolinas@chv.cat) |
| 081885 | Igualada Hospital, Anoia Health Consortium, Igualada | Sergi Sánchez Cordero (PI)  e-mail: [ssanchezco@csa.cat](mailto:ssanchezco@csa.cat)  David Salazar Terceros (Co-PI)  e-mail: dsalazar@csa.cat |
| 430094 | Sant Joan de Reus University Hospital, Reus | Esther Raga Carceller (PI)  e-mail: [estherragacarceller@hotmail.com](mailto:estherragacarceller@hotmail.com) |
| 200185 | Alto Deba Hospital, Mondragon,  San Sebastián | Miguel Calle Baraja (PI)  e-mail: miguel.callebaraja@osakidetza.eus |
| 200163 | Bidasoa Hospital,  San Sebastián | Eulogio Gardeazabal Uriarte (PI)  e-mail: eulogio.gardeazabaluriarte@osakidetza.eus |
| 350228 | Dr.José Molina Orosa Hospital, Lanzarote | Laura Millán Paredes (PI)  e-mail: lmilpar@gobiernodecanarias.org |

*National Catalog of Hospitals (CNH) 2019, Ministry of Health, Consumption and Social Welfare, Spain.

Supplementary data file 1. List of surgical teams proposed to participate in the study *(“COVID-CIR Collaborative Group”)*.
