# Supplementary material for: Postoperative complications and mortality following emergency digestive surgery during the COVID-19 pandemic: A multicenter collaborative retrospective cohort study protocol (COVID-CIR)
Source: Medicine (Baltimore). 2021 Feb 5;100(5):e24409. doi: 10.1097/MD.0000000000024409 (PMC7870207; doi:10.1097/MD.0000000000024409)
Supplement: Supplemental Digital Content [file medi-100-e24409-s002.docx]

| **Data collection**  **instrument** | ***Admission***  ***(dd-mm-yy)*** | ***Preoperative period*** | ***Surgery***  ***(dd-mm-yy)*** | ***Postoperative course*** | ***Follow up*** |
| --- | --- | --- | --- | --- | --- |
| **Inclusion and exclusion criteria** | 2 inclusion criteria (+)  2 exclusión criteria (-) |  |  |  |  |
| **Demographic data** | -admission date  -cause for admission  -date of birth  -age  -sex  -weight  -height  -BMI |  |  |  |  |
| **Clinical condition** | -ASA scale  -functional status  -respiratory system  -cardiac system  -hypertension  -DM  -smoking  -COPD  -cardiovascular history  -preoperative SARS-CoV-2 |  |  |  |  |
| **Preoperative ICU** |  | NO/YES |  |  |  |
| **Vital signs** |  | -temperature  -SBP  -HR  -GCS |  |  |  |
| **Preoperative analysis** |  | -analysis date  -values: Na+, K+, urea, ALT, Hb, leukocytes, neutrophils, lymphocytes, platelets, NLR, PLR, CRP, D-dimer, ferritin, PCT, LDH, TN, PT |  |  |  |
| **EKG** |  | cardiac rhythm |  |  |  |
| **Surgical data** |  |  | -date of surgery  -type of surgery (urgent *vs.* emergency)  -surgical approach (open *vs.* laparoscopy)  -diagnosis  -degree of malignancy  -peritoneal soiling  -estimated bleeding  -primary procedure  -surgical complexity  -associated procedure |  |  |
| **Scores** |  |  |  | -POSSUM (morbidity and mortality)  -P-POSSUM  -LUCENTUM-logistic regression  -LUCENTUM-CHAID |  |
| **Complications**  **≤30 days** |  |  |  | -postoperative SARS-CoV-2 status  -complications (NO/YES)   - selection (30 options) - severity (Clavien-Dindo scale) - related to COVID-19 (NO/YES)   -postoperative ICU/PSR unit admission (NO/YES) |  |
| **Hospital stay** |  |  |  | -exitus during admission (NO/YES)  -discharge (NO/YES)  -date of discharge/death  -length of stay (days) |  |
| **30 days follow up** |  |  |  |  | -readmission (NO/YES)  -reintervention (NO/YES)  -global condition (resolved/sequelae/exitus):   - type of sequelae - exitus date |
| **90 days follow up** |  |  |  |  | -global condition (resolved/sequelae/exitus):   - type of sequelae - exitus date |

dd-mm-yy: "day-month-year" date format; BMI: body mass index; ASA: American Society of Anaesthesiologists score; DM: diabetes; COPD: chronic obstructive pulmonary disease; ICU: Intensive Care Unit; SBP: systolic blood pressure; HR: heart rate; GCS: Glasgow Coma Score; ALT: alanine-aminotransferase; Hb: hemoglobin, NLR: neutrophil/lymphocyte ratio; PLR; platelet/lymphocyte ratio; CRP: C-reactive protein; PCT: procalcitonin; LDH: lactate-dehydrogenase; TN: troponin; PT: prothrombin time; EKG: electrocardiogram; PSR unit: post-surgical reanimation unit. Values marked in red will be automatically calculated by the software *(REDCap database)*.

Supplementary data file 2. eCRF variables by category.
