# Supplementary material for: Postoperative complications and mortality following emergency digestive surgery during the COVID-19 pandemic: A multicenter collaborative retrospective cohort study protocol (COVID-CIR)
Source: Medicine (Baltimore). 2021 Feb 5;100(5):e24409. doi: 10.1097/MD.0000000000024409 (PMC7870207; doi:10.1097/MD.0000000000024409)
Supplement: Supplemental Digital Content [file medi-100-e24409-s003.docx]

Supplementary data file 3. Items listed in the electronic Case Report Form (eCRF).

1- Patient numerical code assignment [automatic]

**DATA ON ADMISSION**

2- Age: [numeric without decimals]

3- Sex: [options]

- Man
- Woman

4- Weight (kg): [numeric with one decimal]

5- Height (m): [numeric with two decimals]

6-Body Mass Index (BMI) (kg/m^2^): [numeric with one decimal] [automatic calculation]

7- ASA (American Society of Anesthesiologists) grade: [options]

- I: Absence of systemic disease
- II: Mild systemic disease
- III: Severe systemic disease affecting patient activity, but not incapacitating
- IV: Incapacitating severe disease, but patient not dying
- V: Moribund patient, not expected to survive 24 hours

8- Functional status: the best functional status of patient autonomy during the 30 days prior to surgery: [options]

• Independent: The patient does not require assistance from another person for any daily activity. Includes people who operate independently with prosthetics or appliances.

• Partially dependent: The patient needs help from another person to perform daily activities.

• Fully dependent: The patient requires full assistance for all activities of daily life.

9- Respiratory system: [options]

- No dyspnea, chest X-ray without COPD (chronic obstructive pulmonary disease) data
- Exercise dyspnea, minimum COPD in chest X-ray
- Limiting dyspnea (1 landing), moderate COPD in chest X-ray
- Dyspnea at rest (≥30 breaths/minute), fibrosis or consolidation in chest X-ray

10- Heart system: [options]

- No failure
- Diuretics, digoxin, antianginal or antihypertensive drugs
- Peripheral edemas, warfarin, incipient cardiomegaly
- Elevated jugular venous pressure, cardiomegaly

11- Date of hospital admission: [date in format days/month/year]

12- Reason for admission: [options]

- COVID-19 infection treatment, no current surgical pathology
- Surgical pathology, no COVID-19 infection clinic
- Surgical pathology and COVID-19 infectious clinical condition
- Another cause

13- Is the patient hypertensive with drug treatment?: [yes/no]

14- Is the patient diabetic with drug treatment?: [yes/no]

15- Is the patient an active smoker?: [yes/no]

16- Is the patient COPD?: [yes/no]

17- Does the patient have a history of ischemic heart disease, cerebrovascular accident (transient ischemic attack, stroke) or peripheral artery disease?: [yes/no]

18- **Preoperative** diagnosis of COVID-19 infection: [options]:

- PCR-positive (RT-Polymerase Chain Reaction, nasopharyngeal sample)
- Serology-positive (rapid test in capillary blood or serological test in laboratory after venous extraction by venipuncture)
- Considered COVID-19-positive by clinical and/or radiological suspicion (chest X-ray, chest computed tomography, thoracic ultrasound)
- Considered COVID-19-negative in the preoperative period

**PREOPERATIVE DATA** (closest to urgent surgery)

19- Intensive Care Unit (ICU) admission before surgery: [yes/no]

20- Reason for ICU entry prior to surgery: [options]

- COVID-19 infection treatment, no current surgical pathology
- Surgical pathology, no COVID-19 infection clinic
- Surgical pathology and clinical pathology of COVID-19 infection
- Other cause

21- Temperature (ºC): [numeric with one decimal]

22- Systolic blood pressure (mmHg): [options]

- 110-129
- 130-170 or 100-109
- <170 or 90-99
- <90

23- Heart rate (beats/minute): [options]

- 50-80
- 81-100 or 40-49
- 101-120
- >120 or <40

24- Glasgow Coma Scale score: [options]

- 15
- 12 to 14
- 9 to 11
- ≤ 8

25- Sodium (mmol/L): [options]

- >136
- 131-135
- 126-130
- <126

26- Potassium (mmol/L): [options]

- 3,5-5
- 3,1-3,4 or 5,1-5,3
- 2,9-3,1 or 5,4-5,9
- <2,9 or >5,9

27- Uremia (mmol/L): [options]

- <7,5
- 7,5-10
- 10,1-15
- >15

28- Alanine-aminotransferase (ALT) (UI/L): [options]

- ≤50
- >50

29- Hemoglobin (g/dL): [options]

- 13-16
- 11,5-12,9 or 16,1-17
- 10-11,4 or 17,1-18
- <10 or >18

30- Leukocytes (x10^9^/L): [options]

- 4-10
- 10,1-20 or 3,1-3,9
- >20 or <3,1

31- Neutrophils (x10^9^/L): [numeric with one decimal]

32- Lymphocytes (x10^9^/L): [numeric with one decimal]

33- Platelets (x10^9^/L): [numeric without decimal]

34- NLR (Neutrophil-to-Lymphocyte ratio): ratio between neutrophil figure and lymphocyte figure: [numeric with one decimal] [automatic calculation]

35- PLR (Platelet-to-Lymphocyte ratio): ratio between platelet count and lymphocyte figure: [numeric with one decimal] [automatic calculation]

36- C-reactive protein (mg/L): [numeric with one decimal]

37- D-dimer (μg/L): [numeric without decimals]

38- Ferritin (μg/L): [numeric with one decimal]

39- Procalcitonin (ng/mL, μg/L): [numeric with one decimal]

40- Lactate-dehydrogenase (LDH) (U/L): [numeric without decimals]

41- Troponin (ng/L): [numeric without decimals]

42- Prothrombin time (expressed as a percentage [%], seconds or Quick value): [numeric with one decimal, indicating unit of measurement]

43- E.K.G. (electrocardiogram): [options]

- Normal
- Controlled atrial fibrillation at 60-90 beats/minute
- Any other arrhythmia, ≥5 ventricular extrasystoles/minute, Q-waves or changes in S-T segment or in T-wave.

**OPERATIVE DATA**

44- Date of surgery: [date in day/month/year format]

45- Type of urgent surgery: [options]

- Urgent (>2 hours, <24 hours from indication)
- Emergency (<2 hours from indication)

46. Surgical approach: [options]

- Open
- Laparoscopic

47- Diagnosis: [free text]

48- Malignancy: [options]:

- No
- Localized cancer
- Nodal metastasis (adenopathy)
- Metastasis/disseminated neoplasia
- Report the following cancers as "disseminated": acute lymphocytic leukemia, acute myelocytic leukemia and stage IV lymphoma.
- Mark as presence of "adenopathies": chronic lymphocytic leukemia, chronic myelocytic leukemia, stage <IV lymphomas and multiple myeloma.

49- Peritoneal exudate: [options]

- None
- Serous
- Located pus
- Diffuse peritonitis

50- Blood loss (mL): [options]

- < 100
- 101-500
- 501 – 1000
- 1000

51- Surgical procedure(s) [see Table 1]: [options, possibility of marking more than one option]

Minor complexity:

- - Hernia/eventration, perianal surgery, *sinus* pilonidal

Moderate complexity

- - Cholecystectomy (open or laparoscopic)
  - Appendectomy (open or laparoscopic)

Major complexity

- - Gastrointestinal perforation suture
  - Intestinal resection
  - Colectomy
  - Main bile duct surgery
  - Gastrectomy
  - Lysis of adhesions or reduction of internal hernia or enterolithotomy
  - Splenectomy or minor liver trauma
  - Exploratory laparotomy
  - Surgical hemostasis due to hemoperitoneum

Major + complexity

- - Open pancreatic necrosectomy
  - Pancreatectomy
  - Damage control surgery (due to trauma, bleeding, ischemia or peritonitis)
  - Others: [free text]

52- Surgical pathology related to COVID infection (or treatment): [options]

- No
- Possible
- Probable

53- Explanation of possible relationship: [free text]

54- Do you think that the time of indication of surgery was different from that of the "usual practice" for this pathology?: [options]

- No
- The patient probably consulted later than usual (more advanced pathology)
- The diagnosis of surgical pathology was probably late (diagnostic delay) or urgent intervention was excessively delayed due to logistical problems (hospital delay)
- In this case, conservative treatment (non-operative management) was probably "over-indicated"

55- Do you think that the surgical technique used was different from the "usual practice" for this pathology?: [options]

- No
- Surgical technique was probably more conservative than usual
- Surgical technique was probably more aggressive than usual

**POSTOPERATIVE COURSE**

56- Postoperative complications (≤30 days from the date of urgent intervention): [yes/no]

57- **Postoperative** diagnosis of COVID-19 infection (only in patients not diagnosed preoperatively as COVID-19-positive): [options]

- PCR-positive (nasopharyngeal sample)
- Serology-positive
- Considered COVID-19-positive by clinical and/or radiological suspicion (chest X-ray, chest computed tomography, thoracic ultrasound)
- Considered COVID-19-negative in the postoperative period

58- Type of postoperative complication [see Table 2]: [options, possibility of marking more than one option]

- Anastomotic dehiscence/intestinal fistula: clinical or radiological data of extravasation of intestinal content through an anastomosis, drainage, surgical wound, or abnormal hole
- Superficial wound dehiscence (full fascia)
- Evisceration (clinical or radiological; includes any degree)
- Heart failure or acute pulmonary edema: symptoms or signs of left or congestive ventricular insufficiency (change from preoperative situation)
- Fever of unknown origin: maintained fever >37ºC for ≥24 hours, of unknown cause, after usual immediate postoperative temperature increase
- Mild hemorrhage: post-operative hemorrhage that does not require surgical re-examination or endoscopic/radiological procedure for treatment
- Severe bleeding: post-operative hemorrhage that does require surgical re-examination or endoscopic/radiological procedure for treatment
- Hypotension: mantained drop of systolic pressure to <90 mmHg for > 24 hours, detected with sphygmomanometer or arterial catheter
- Superficial wound infection: redness and pain around the surgical wound or local suppuration
- Deep wound infection: intra-abdominal collection (abscess), clinically or radiologically confirmed, or release of purulent content through a drainage
- Respiratory infection or pneumonia: purulent sputum with positive bacteriological/virologic culture, with or without changes in chest X-ray, or fever with pulmonary radiological consolidation
- Urinary tract infection: urinary symptoms or fever, associated with sediment with bacteriuria/leukocyturia or positive urine culture
- Renal failure: sharp increase in creatinine to ≥2 mg/dL (≥177 μmol/L) in patients with normal prior renal function, or a sharp increase in creatinine (>50%) in patients with chronic kidney failure, or need for renal replacement therapy
- Respiratory failure: breathing difficulty requiring emergency ventilatory support, or PaO_2_ <60 mmHg and PaCO_2_> 45 mmHg breathing ambient air
- Bacteremia-Sepsis: positive blood culture
- DVT (deep vein thrombosis) and/or PE (pulmonary embolism): clinical suspicion, radiological confirmation by ECO-doppler, chest-computed tomography or ventilation/perfusion scan, or *post-mortem* diagnosis
- Postoperative ileus
- Pleural effusion/pulmonary atelectasis
- Intestinal perforation
- Seroma or surgical wound hematoma
- Intestinal occlusion
- Ostomy complications (bleeding, retraction, infection, dermatitis, fistula, stoma necrosis)
- Blood glucose disturbances maintained >24 hours
- Atrial fibrillation
- Hypertensive crises (systolic blood pressure >200 mmHg and/or diastolic blood pressure >120 mmHg)
- Acute confusion syndrome
- Gastrointestinal bleeding (upper or lower)
- Acute myocardial infarction, cerebrovascular accident or acute limb ischemia (peripheral artery ischemia)
- Acute mesenteric ischemia (intestinal ischemia, small bowel or colon)
- Cardiomyopathy or pericarditis

59- Severity of complication (Clavien-Dindo classification; choosing the most serious complication): [options]

- No complications.
- I: Any deviation from the normal postoperative course that does not require surgical, radiological, or endoscopic reintervention. Includes additional use of electrolyte solutions, diuretics, antiemetics, antipyretics, analgesics, and physiotherapy. Includes superficial infection or seroma/hematoma treated at the bedside.
- II: Different pharmacological treatment than the above ones is required, including blood transfusions, antibiotics or total parenteral nutrition.
- III: Surgical, endoscopic, or radiological intervention is required.
  - IIIa: No general anesthesia
  - IIIb: Under general anesthesia
- IV: Life-threatening complications requiring treatment in intermediate or intensive care unit:
  - IVa: Single organ dysfunction (includes hemodialysis)
  - IVb: Multiple organic dysfunction
- V: Patient death

60-The patient has developed some postoperative complication (≤30 days) characteristic of COVID-19 infection: [yes/no]

61-Were ICU or Post-Surgical Reanimation unit required (for >24 hours) after the first urgent surgery?: [yes/no]

62-Was any surgical reintervention required within 30 days of the first urgent surgery?: [yes/no]

63- Date of hospital discharge (or death during hospital stay): [date]

64- Length of hospital stay (up to discharge or death): [numeric with one decimal] [automatic calculation]

65- Hospital readmission in the first **30 days** after discharge: [yes/no]

66- Current patient status **30 days** after urgent operation: [options]

- Resolved
- Pending sequels
- Permanent sequels
- Exitus

67- Current patient status **90 days** after urgent operation: [options]

- Resolved
- Pending sequels
- Permanent sequels
- Exitus

68- Description of the sequels: [free text]

69- Date of exitus: [date in day/month/year format]
